# A Comparison of Classical and Modern Measures of Internal Consistency

**DOI:** 10.3389/fpsyg.2019.02714

**Published:** 2019-12-04

**Authors:** Pasquale Anselmi, Daiana Colledani, Egidio Robusto

**Affiliations:** Department of Philosophy, Sociology, Education and Applied Psychology, University of Padua, Padua, Italy

**Keywords:** internal consistency, reliability, Rasch models, modern test theory, classical test theory, infit, outfit

## Abstract

Three measures of internal consistency – Kuder-Richardson Formula 20 (KR20), Cronbach’s alpha (α), and person separation reliability (R) – are considered. KR20 and α are common measures in classical test theory, whereas R is developed in modern test theory and, more precisely, in Rasch measurement. These three measures specify the observed variance as the sum of true variance and error variance. However, they differ for the way in which these quantities are obtained. KR20 uses the error variance of an “average” respondent from the sample, which overestimates the error variance of respondents with high or low scores. Conversely, R uses the actual average error variance of the sample. KR20 and α use respondents’ test scores in calculating the observed variance. This is potentially misleading because test scores are not linear representations of the underlying variable, whereas calculation of variance requires linearity. Contrariwise, if the data fit the Rasch model, the measures estimated for each respondent are on a linear scale, thus being numerically suitable for calculating the observed variance. Given these differences, R is expected to be a better index of internal consistency than KR20 and α. The present work compares the three measures on simulated data sets with dichotomous and polytomous items. It is shown that all the estimates of internal consistency decrease with the increasing of the skewness of the score distribution, with R decreasing to a larger extent. Thus, R is more conservative than KR20 and α, and prevents test users from believing a test has better measurement characteristics than it actually has. In addition, it is shown that Rasch-based infit and outfit person statistics can be used for handling data sets with random responses. Two options are described. The first one implies computing a more conservative estimate of internal consistency. The second one implies detecting individuals with random responses. When there are a few individuals with a consistent number of random responses, infit and outfit allow for correctly detecting almost all of them. Once these individuals are removed, a “cleaned” data set is obtained that can be used for computing a less biased estimate of internal consistency.

## Introduction

The present work deals with internal consistency, which expresses the degree to which the items of a test produce similar scores. Three measures of internal consistency are considered, namely Kuder-Richardson Formula 20 (KR20; Kuder and Richardson, 1937), Cronbach’s α (Cronbach, 1951), and person separation reliability (R; Wright and Masters, 1982).

KR20 and α are well-known measures in classical test theory, where they are widely used to evaluate the internal consistency of cognitive and personality tests. The derivations of KR20 and α used continuous random variables for item scores (Sijtsma, 2009). As such, they include dichotomous scoring (e.g., correct/incorrect; yes/no) and ordered polytomous scoring (e.g., never/sometimes/often/always; very difficult/difficult/easy/very easy) as special cases. The formula for the computation of KR20 is suitable for items with dichotomous scores, whereas the formula for the computation of α is suitable for items with dichotomous scores and items with polytomous scores. When all items are scored 1 or 0, the formula for KR20 reduces to that for α (Cronbach, 1951).

Less known than KR20 and α, R develops within modern test theory and, more precisely, within Rasch models. There are several applications of these models to the development and validation of measurement instruments (see, e.g., Duncan et al., 2003; Cole et al., 2004; Vidotto et al., 2006, 2007, 2010; Pallant and Tennant, 2007; Shea et al., 2009; Anselmi et al., 2011, 2013a,b, 2015; Da Dalt et al., 2013, 2015, 2017; Balsamo et al., 2014; Liu et al., 2017; Rossi Ferrario et al., 2019; Sotgiu et al., 2019). Rasch models characterize the responses of persons to items as a function of person and item measures (in the Rasch framework, the terms “person measure” and “item measure” are used to denote the values of the person parameter and item parameter, respectively). These measures pertain to the level of a quantitative latent trait possessed by a person or item, and their specific meaning relies on the subject of the assessment. In educational assessments, for instance, person measures indicate the ability of persons, and item measures indicate the difficulty of items. In health status assessments, person measures indicate the health of persons, and item measures indicate the severity of items. The Rasch model for dichotomous items is the simple logistic model (SLM; Rasch, 1960). This model allows for estimating a measure for each person and a measure for each item. An extension of the SLM to polytomous items is the rating scale model (RSM; Andrich, 1978). In addition to the measures estimated by the SLM, the RSM also estimates measures that describe the functioning of the response scale. These measures, called thresholds, represent the point on the latent variable where adjacent response categories are equally probable. If the thresholds are increasingly ordered, then the response scale functions as expected (i.e., increasing levels of the latent variable in a respondent correspond to increasing probabilities that the respondent will choose the higher response categories; Linacre, 2002a; Tennant, 2004). R can be computed both for the person measures estimated on dichotomous data and for the person measures estimated on polytomous data.

KR20, α, and R are based on the essentially tau-equivalent measurement model, a measurement model that requires a number of assumptions to be met for the estimate to accurately reflect the true reliability. Essential tau-equivalence assumes that each item measures the same latent variable (unidimensionality), on the same scale (similar variances), but with possibly different degrees of precision (different means; Raykov, 1997). Within the framework of factor analysis, essential tau-equivalence is represented by all items having equal factor loadings on a single underlying factor (McDonald, 1999). Graham (2006) provides a nice example to describe this measurement model. The author considers a test designed to measure depression in which each item is measured on a five-point Likert scale from “strongly disagree” to “strongly agree.” Responses to items like “I feel sad sometimes” and “I almost always feel sad” are likely to have similar distributions, but with different modes. This might be due to the fact that, though both items measure the same latent variable on the same scale, the second one is worded more strongly than the first. As long as the variances of these items are similar across respondents, they are both measuring depression in the same scale, but with different precision.

KR20, α, and R are all estimates of the ratio between true variance and observed variance, and specify the observed variance as the sum of true and error variance. However, they differ for the way in which these quantities are obtained. Let us consider, for instance, a cognitive test with correct and incorrect item responses. In KR20, the error variance is computed as the sum of the variances of the items. In particular, with *p*_*i*_ denoting the proportion of correct responses to item *i* = 1,2,…,*I*, the error variance is $\sum_{i=1}^{I}p_{i}\,\left(1-p_{i}\right)$. For dichotomous responses, *p*_*i*_ corresponds to the sample mean of the responses to item *i*. Thus, it represents what is expected from an “average” respondent from the sample on item *i* (Wright and Stone, 1999). When the variances *p*_*i*_(1−*p*_*i*_) are summed across the items, an error variance is obtained that represents the error variance of an “average” respondent from the sample. Respondents with high or low scores have less error variance than “average” respondents. Thus, the error variance of an “average” respondent used in KR20 overestimates the error variance of respondents with high or low scores. Furthermore, such an error variance is not the same as an average of the error variances of individual respondents. If the score distribution is not symmetric, the two quantities are different (Wright and Stone, 1999). Rasch measurement provides, for each estimate of a respondent’s trait level, an accompanying estimate of the precision of the measure, called standard error (*SE*). The lower the *SE*, the higher the precision of trait level estimate. These individual *SE*s are used to compute the average error variance of the sample. In particular, with *SE*_*n*_ denoting the standard error associated with the trait level estimate of respondent *n* = 1, 2, …, *N*, the average error variance of the sample is given by $\frac{\sum_{n=1}^{N}S\,E_{n}^{2}}{N}$.

KR20 and α use respondents’ test scores (each of which being the sum of the responses over all items) in calculating the observed variance. This is potentially misleading. On the one hand, test scores are not linear representations of the variable they are intended to represent. For instance, a compression of the scale is bound to occur near the lower and upper boundaries of the score domain (“floor” and “ceiling” effects, respectively; Fischer, 2003). On the other hand, calculation of mean and variance necessary to obtain the observed variance assumes linearity in the numbers that are used (Wright and Stone, 1999). Thus, the observed variance computed from test scores might be incorrect to some degree. Contrariwise, if the data fit the Rasch model, the measures estimated for each respondent are on a linear scale, thus being numerically suitable for calculating the observed variance (Wright and Stone, 1999; Smith, 2001).

Given the aforementioned differences, classical and modern estimates of internal consistency might differ to some extent. Compared with KR20 and α, R is expected to be a better index of internal consistency as the numerical values are linear rather than non-linear, and the actual average error variance of the sample is used instead on the error variance of an “average” respondent.

The estimates of internal consistency might be affected by particular response behaviors. For instance, Pastore and Lombardi (2013) observed that α decreases with the increasing of the proportion of fake-good responses (i.e., responses aimed at providing a positive self-description) in the data set. The estimates of internal consistency might also be affected by random responding, that is a response set where individuals do not consider the content of the items and randomly choose all response options one by one. Random responding is not uncommon when respondents do not have an intrinsic interest in the investigation, the test is long, and the setting is uncontrolled (such as, e.g., in interned-based surveys; Johnson, 2005; Meade and Craig, 2012).

A method for identifying random responding requires the use of special items and scales. Examples include bogus items (e.g., “the water is wet”), instructed response items (e.g., “respond with a 2 for this item”), lie scales (e.g., MMPI-2 Lie scale), and scales for assessing inconsistent responding (e.g., MMPI-2 VRIN and TRIN scales). A drawback of this method is that testing time is lengthened.

Rasch framework provides methods and procedures for identifying and handling unexpected response behaviors. Mean-square fit statistics are computed for each individual and each item. Their expected value is 1. Values greater than 1 indicate underfit to the model (i.e., the responses are less predictable than the Rasch model expects), whereas values smaller than 1 indicate overfit (i.e., the responses are more predictable than the model expects; Linacre, 2002b). There are two types of mean-square fit statistics: outfit and infit. Outfit is mostly influenced by unexpected responses of high entity, whereas infit is mostly influenced by unexpected responses of small entity. An example of unexpected response is an incorrect response to an item for which a correct response is expected (i.e., an item for which, according to the Rasch model, the probability of a correct response is larger than that of an incorrect response). If the probability of the correct response is much larger than that of the incorrect response, the unexpected response mainly influences outfit. If the probability of the correct response is slightly larger than that of the incorrect response, the unexpected response mainly influences infit.

Infit and outfit allow for detecting individuals with unexpected response behaviors. For instance, they have been used to identify possible fakers to self-report personality tests (Vidotto et al., 2018) and to identify individuals who miss responses to items they are not capable of solving (Anselmi et al., 2018). In the present work, infit and oufit are used for handling random responses in the estimation of internal consistency. Two options are available. The first option implies taking into account random responses in order to compute a more conservative estimate of internal consistency. In the Rasch framework, this is done by enlarging the *SE* of latent trait estimates of those individuals with infit statistic larger than 1. With *SE*_*n*_ denoting the standard error associated with the trait level estimate of respondent *n* = 1, 2, …, *N*, and infit*_*n*_* denoting his/her infit statistic, the new infit-inflated standard error is given by *SE*_*n*_ × max(1, infit*_*n*_*) (see, e.g., Linacre, 1997). Then, this new standard error is used in place of *SE*_*n*_ to compute the average error variance of the sample. In the present work, a modification of this procedure is presented, in which an outfit-inflated standard error is computed as *SE*_*n*_ × max(1, outfit*_*n*_*). The larger the percentage of random responses, the larger the infit/outfit-inflated standard errors and the lower the estimate of internal consistency.

The second option implies “cleaning” the data set before estimating internal consistency. To this aim, individuals with infit or outfit above a certain, appropriately chosen cut-off are flagged as possible respondents with random responses and removed. A conservative choice for the cut-off is 1.3 (Wright and Linacre, 1994). Such a value indicates that, in the response pattern, there is 30% more randomness than expected by the Rasch models. If most individuals with random responses are correctly identified and removed, the internal consistency estimated on the “cleaned” data set should be less biased than that estimated on the “uncleaned” data set.

The aim of the present work is twofold. Firstly, it attempts to show the conditions in which classical and modern estimates of internal consistency are similar and those in which they are not. To this aim, data sets are simulated that differ for the distribution of test scores. Secondly, it investigates the use of respondents’ infit and outfit statistics to compute more conservative estimates of internal consistency or to detect individuals with random responses. To this aim, data sets are simulated that include different percentages of random responses. Tests with dichotomous items and tests with polytomous items are considered.

## Study 1 – Effects of Score Distribution on Internal Consistency Measures

The present study aims at investigating the effects of score distribution on classical and modern estimates of internal consistency. Data sets are simulated that differ for the skewness of the score distribution. Classical and modern measures are expected to be substantially the same when the score distribution is symmetric, whereas they are expected to differ more and more with the increasing of the skewness of the score distribution. This study largely resembles that described by Linacre (1997). The author has only dealt with the dichotomous case and generated a single data set for each skewness condition. In the present study, both the dichotomous and polytomous cases are considered, and multiple data sets are generated for each skewness condition.

### Data Simulation

All the data sets simulated in this study consist of the responses of 100 individuals to tests with 30 items. The polytomous data sets were simulated considering items with four response categories. Different skewed score distributions were obtained using the following three-step procedure:

1. A total of 30 true item measures were randomly drawn from a uniform distribution defined on the interval [−3, 3]. When simulating the polytomous data, three true thresholds were randomly simulated (i.e., the threshold between responses 1 and 2, that between 2 and 3, and that between 3 and 4) that were increasingly ordered and equally distant from each other. A total of 100 true person measures were randomly drawn from a standard normal distribution. This construction results in a sample of simulated respondents that is targeted on the test. This condition is denoted with offset = 0.
2. Four mistargeted samples were obtained by adding one, two, three, or four logits to the true person measures drawn in Step 1 (the logits are the measurement units constructed by Rasch models; Wright, 1993). These conditions are denoted with offset = 1, 2, 3, and 4.
3. Data sets were simulated for each of the five offset conditions. The dichotomous data sets were simulated using the SLM (Rasch, 1960), whereas the polytomous data sets were simulated using the RSM (Andrich, 1978).

It is noted in passing that the use of a uniform distribution for the item measures is a common choice (Linacre, 2007), and depicts the condition of tests measuring the different latent trait levels with the same precision. The use of thresholds that are increasingly ordered and equally distant depicts the condition of a well-functioning response scale (i.e., the response options are equally relevant and their choice appropriately reflects respondents’ latent trait levels).

The aforementioned three-step procedure was repeated 100 times. Thus, 100 data sets were simulated for each of five offset conditions.

### Results

**FIGURE 1:**
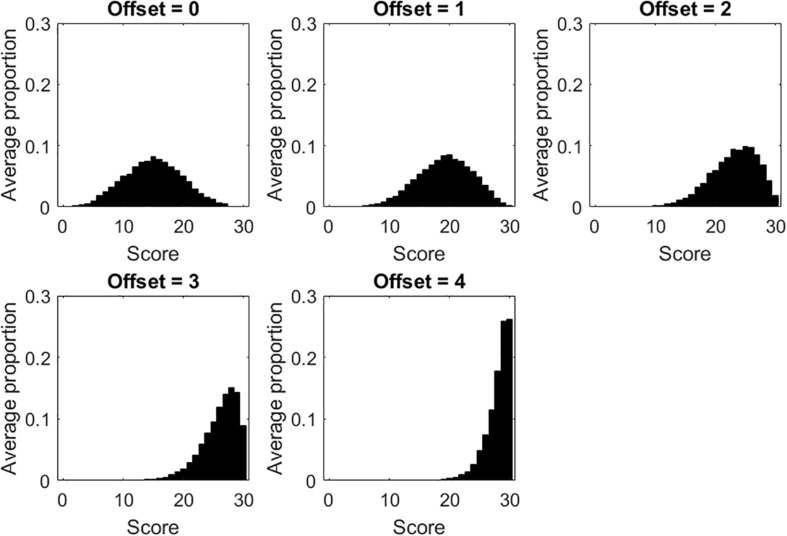
Score distributions for each of the five offset conditions in the tests with dichotomous items.

Results considering the tests with dichotomous items are considered first. For each of the five offset conditions, Figure 1 displays the score distribution, averaged across the 100 data sets simulated for that condition. When offset = 0 (i.e., the sample is targeted on the test), the score distribution resembles the distribution of person measures. Contrariwise, as offset increases (i.e., the samples are less and less targeted on the tests), the score distributions are more skewed, with high scores becoming more and more frequent. Ceiling effects are observed when offset is 3 or 4. It is worth noting that, in the five offset conditions, the underlying distribution of person measures is always the normal distribution.

**FIGURE 2:**
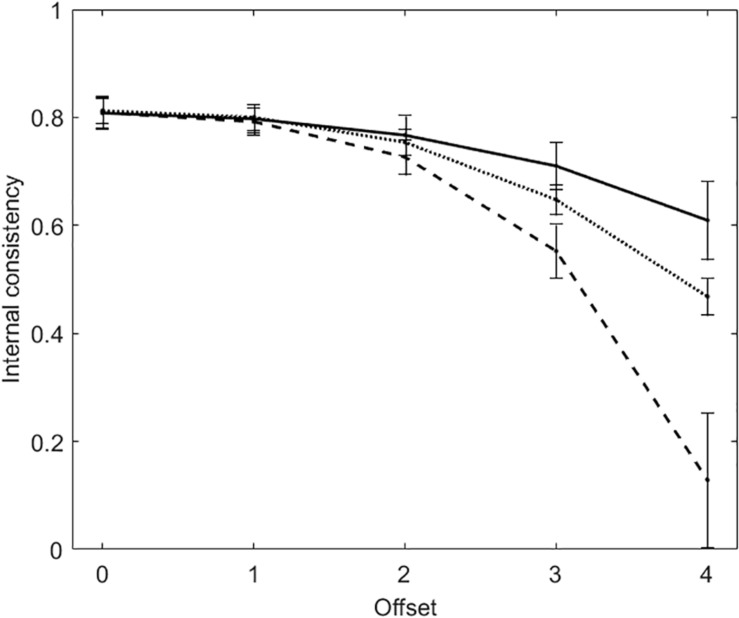
Average internal consistency (and standard deviation) for each of the five offset conditions in the tests with dichotomous items. The solid line represents KR20, the dashed line represents R, the dotted line represents the true-measure-based internal consistency (TMBIC).

Figure 2 plots average internal consistency (and standard deviation) for each of the five offset conditions. There are three lines in the figure. The solid line and the dashed line represent KR20 and R, respectively. The dotted line represents the true-measure-based internal consistency (TMBIC), which is a Rasch measure of internal consistency computed directly from the true person and item measures, without data. In the computation of TMBIC, the true variance is the variance of the true person measures, whereas the *SE*s that are necessary to obtain the error variance are derived from the true person and item measures). TMBIC is taken to be the maximum possible internal consistency under the Rasch model (Linacre, 1997).

When offset = 0, KR20 and R are virtually the same (*M*_KR__20_ = *M*_R_ = 0.81; *SD*_KR__20_ = *SD*_R_ = 0.03). Both the measures of internal consistency decrease with the increasing of offset, with R decreasing to a larger extent. With offset = 3, KR20 suggests that internal consistency is acceptable (*M* = 0.71, *SD* = 0.04), whereas R does not (*M* = 0.55, *SD* = 0.05). KR20 is larger than TMBIC, whereas R is smaller.

**FIGURE 3:**
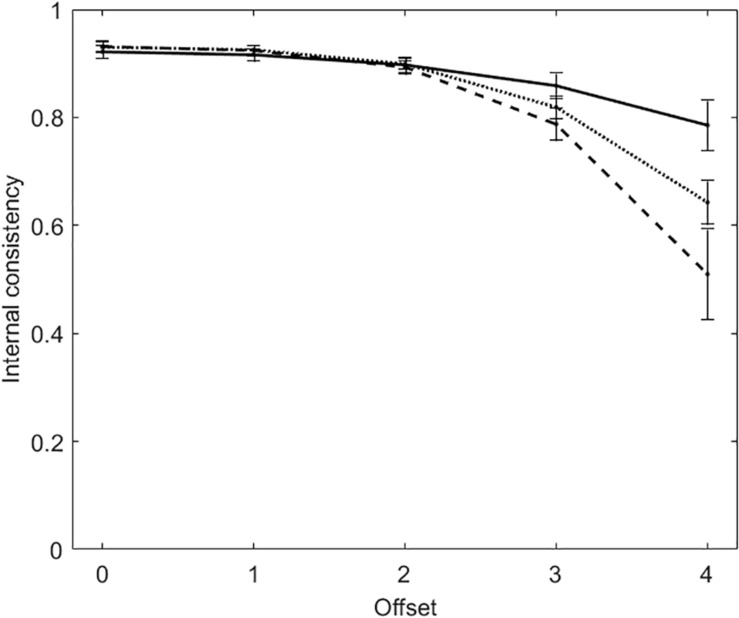
Average internal consistency (and standard deviation) for each of the five offset conditions in the tests with polytomous items. The solid line represents α, the dashed line represents R, the dotted line represents the true-measure-based internal consistency (TMBIC).

Also in the tests with polytomous items, the score distributions become more and more skewed with the increasing of offset. Figure 3 plots α (solid line), R (dashed line), and TMBIC (dotted line) against the five offset conditions. As for the dichotomous tests, the two measures of internal consistency decrease with the increasing of offset. The two measures are largely the same when offset ≤ 2, whereas they differ when offset is 3 or 4. When offset = 4, α suggests that internal consistency is acceptable (*M* = 0.79, *SD* = 0.05), whereas R does not (*M* = 0.51, *SD* = 0.08). In addition, α is larger than TMBIC, whereas R is smaller. Offset being the same, internal consistency is larger in the polytomous tests than in the dichotomous tests. This result is due to the fact that, the number items being equal, internal consistency increases with the number of response categories (Lozano et al., 2008).

### Brief Discussion

When the score distributions are substantially symmetric, classical and modern estimates of internal consistency are largely the same. In the case of a symmetric score distribution, the error variance estimated by KR20 and α largely resembles that resulting from R. Moreover, in the middle of the score domain, the relationship between scores and measures is approximately linear. Thus, when the largest part of the scores belongs to this central region (as it is in a symmetric score distribution), the observed variance obtained from scores is similar to that obtained from measures.

In presence of skewed score distributions, classical and modern estimates of internal consistency differ. Andrich (2016) warns researchers that “distributions skewed artificially because of floor or ceiling effects render the calculation of α essentially meaningless” (Andrich, 2016, p. 29). It is worth noting that R is more conservative than KR20 and α. In addition, R is lower than TMBIC, whereas KR20 and α are larger. Thus, using R in place of the classical measures reduces the changes of test users attributing the test better measurement characteristics than it actually has.

The dichotomous and polytomous tests are not directly comparable, even if they contain the same number of items. This is due to the fact that internal consistency increases not only with the number of items but also with the number of response categories (Lozano et al., 2008). To this respect, a test with 30 polytomous items each having four response categories is analogous to a test with 90 dichotomous items. Similarly, a test with 30 dichotomous items is analogous to a test with 10 items each having four response categories. This explains why, offset being the same, internal consistency was larger in the polytomous tests than in the dichotomous tests.

## Study 2 – Handling Unexpected Response Behaviors When Computing Internal Consistency

The present study aims at investigating the use of infit and outfit statistics to compute more conservative estimates of internal consistency and to detect individuals with random responses. Data sets are simulated that differ for (a) the percentage of respondents with random responses, and (b) the percentage of items with random responses. It is expected that, with the increasing of the two percentages, internal consistency decreases. Moreover, it is expected that, if the respondents with random responses are correctly identified and removed, the internal consistency computed on the cleaned data sets is similar to the true internal consistency.

### Data Simulation

All the data sets simulated in this study consist of the responses of 100 individuals to tests with 30 items. The polytomous data sets were simulated considering items with four response categories. The data sets were obtained using the following three-step procedure:

1. A total of 30 true item measures were randomly drawn from a uniform distribution defined on the interval [−3, 3]. When simulating the polytomous data, three true thresholds were randomly simulated that were increasingly ordered and equally distant from each other. A total of 100 true person measures were randomly drawn from a standard normal distribution.
2. Data sets were simulated using the measures drawn in Step 1. The dichotomous data sets were simulated using the SLM (Rasch, 1960), whereas the polytomous data sets were simulated using the RSM (Andrich, 1978).
3. Twenty-five data sets with random responses were obtained from the data sets simulated at Step 2. These data sets differed for the proportion of simulees with random responses (*p*_sim_ = 0.10, 0.20, 0.30, 0.40, 0.50), and for the proportion of random item responses (*p*_resp_ = 0.10, 0.20, 0.30, 0.40, 0.50). The condition with *p*_sim_ = 0.20 and *p*_resp_ = 0.30 indicates 30% of random responses (i.e., 9 items) for 20% of simulees (i.e., 20 simulees). For each simulee, the items with random responses were randomly selected, and the responses to these items were set to be different to those in the original simulated data set.

The aforementioned three-step procedure was repeated 100 times. This resulted in 100 data sets without random responses, and 100 × 25 data sets with random responses (denoted as “uncleaned” data sets).

### Results

#### Computing More Conservative Estimates of Internal Consistency

**FIGURE 4:**
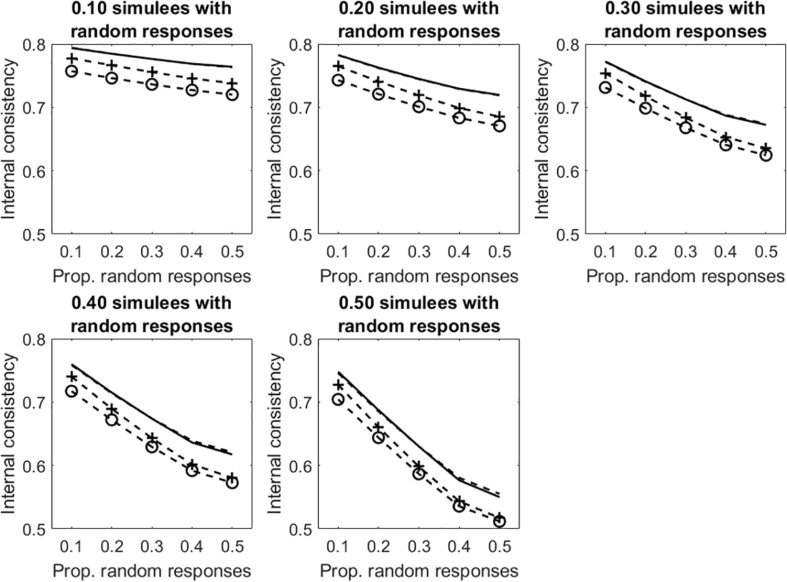
Average internal consistency for the different proportions of simulees with random responses and the different proportions of dichotomous items with random responses. The solid line represents KR20, the unmarked dashed line represents R, the +-marked dashed line represents infit-corrected R, and the o-marked dashed line represents the outfit-corrected R.

Results concerning the tests with dichotomous items are considered first. Figure 4 displays the average internal consistency for the different proportions of simulees with random responses and the different proportions of items with random responses. There are four lines in each panel. The solid line represents KR20, the (unmarked) dashed line represents R, the +-marked dashed line represents infit-corrected R and the o-marked dashed line represents the outfit-corrected R. Some comments to the figure follows. In all the conditions, uncorrected KR20 and R lead to the same measure of internal consistency (the solid line substantially overlaps the unmarked dashed line). As shown in Study 1, when the samples are well-targeted on the tests (as it is in the case considered here), then KR20 and R lead to virtually the same estimate of internal consistency. As expected, all the internal consistency measures decrease with the increasing of the proportion of simulees with random responses and with the proportion of items in the patterns with random responses. The two underfit-corrected R measures of internal consistency (the two marked lines) are systematically lower than the two uncorrected measures (the two unmarked lines). The outfit-corrected R measure of internal consistency (the o-marked dashed line) is systematically lower than the infit-corrected R measure (the +-marked dashed line).

**FIGURE 5:**
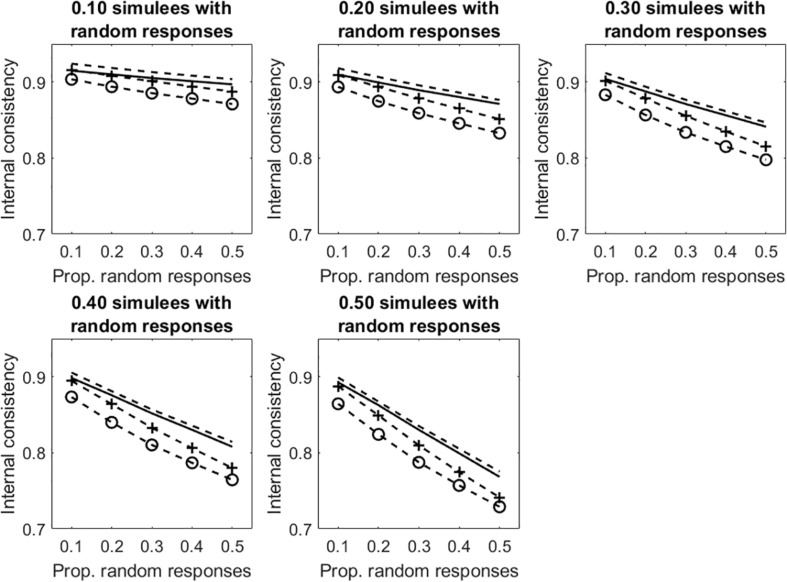
Average internal consistency for the different proportions of simulees with random responses and the different proportions of polytomous items with random responses. The solid line represents α, the unmarked dashed line represents R, the +-marked dashed line represents infit-corrected R, and the o-marked dashed line represents the outfit-corrected R.

Figure 5 depicts the results concerning the tests with polytomous items. Results are similar to those observed in the dichotomous case. Given otherwise identical conditions, internal consistency is systematically larger in the polytomous case than in the dichotomous case. As discussed in Study 1, this result is due to the fact that, the number items being equal, internal consistency increases with the number of response categories.

#### Detection of Simulees With Random Responses

For each data set and each fit statistic (infit, outfit), sensitivity and specificity of the cut-off at 1.3 were computed by creating a 2 × 2 contingency matrix as follows:


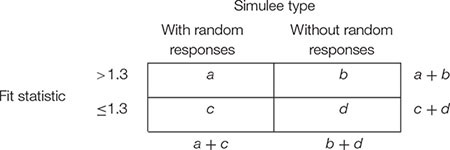


Sensitivity refers to the capacity of correctly detecting simulees with random responses. It is the proportion of simulees with fit statistic larger than 1.3 among those simulees with random responses, that is *a*/(*a* + *c*). Specificity refers to the capacity of correctly ignoring simulees without random responses. It is the proportion of simulees with fit statistic smaller than or equal to 1.3 among those simulees without random responses, that is *d*/(*b* + *d*).

Table 1 shows sensitivity and specificity of infit and outfit statistics in the tests with dichotomous items. Both the proportion of simulees with random responses and the proportion of random responses in the patterns affect sensitivity. Overall, the lower the proportion of simulees with random responses and the higher the proportion of random responses in the patterns, the higher the sensitivity. A cut-off at 1.3 allows for detecting only 13% (infit) or 30% (outfit) of simulees with random responses when these simulees represent 50% of the sample and the random responses concern 10% of the items. Conversely, the same cut-off allows for detecting almost all simulees with random responses when they represent 10% of the sample and the random responses concern 50% of the items (sensitivity = 0.98, 0.99 for infit and outfit, respectively). Sensitivity of the cut-off on outfit is always larger than that of the cut-off on infit. Specificity remains very high regardless of the proportion of simulees with random responses and the proportion of random responses in the patterns (specificity from 0.93 to 1 for infit; from 0.86 to 1 for outfit). Taken all together, these results suggest that, when there are a few individuals with a consistent number of random responses, a cut-off at 1.3 allows for detecting almost all of them.

**TABLE 1:** Sensitivity and specificity of infit and outfit in the tests with dichotomous items.

		**Infit**		**Outfit**	
***p*_sim_**	***p*_resp_**	**Sensitivity**	**Specificity**	**Sensitivity**	**Specificity**
0.10	0.10	0.30	0.93	0.51	0.86
0.10	0.20	0.58	0.95	0.76	0.88
0.10	0.30	0.84	0.96	0.90	0.90
0.10	0.40	0.93	0.97	0.97	0.92
0.10	0.50	0.98	0.98	0.99	0.94
0.20	0.10	0.25	0.95	0.46	0.88
0.20	0.20	0.50	0.98	0.66	0.93
0.20	0.30	0.69	0.99	0.80	0.95
0.20	0.40	0.84	0.99	0.90	0.97
0.20	0.50	0.92	1.00	0.96	0.98
0.30	0.10	0.21	0.97	0.41	0.91
0.30	0.20	0.37	0.99	0.56	0.95
0.30	0.30	0.52	1.00	0.67	0.97
0.30	0.40	0.65	1.00	0.76	0.99
0.30	0.50	0.73	1.00	0.83	1.00
0.40	0.10	0.17	0.98	0.35	0.92
0.40	0.20	0.24	0.99	0.44	0.97
0.40	0.30	0.34	1.00	0.50	0.99
0.40	0.40	0.39	1.00	0.53	1.00
0.40	0.50	0.42	1.00	0.55	1.00
0.50	0.10	0.13	0.98	0.30	0.94
0.50	0.20	0.16	1.00	0.33	0.98
0.50	0.30	0.18	1.00	0.32	1.00
0.50	0.40	0.16	1.00	0.28	1.00
0.50	0.50	0.11	1.00	0.20	1.00

*p_*sim*_ = proportion of simulees with random responses; p_*resp*_ = proportion of random item responses. Cut-off for infit and outfit = 1.3.*

**FIGURE 6:**
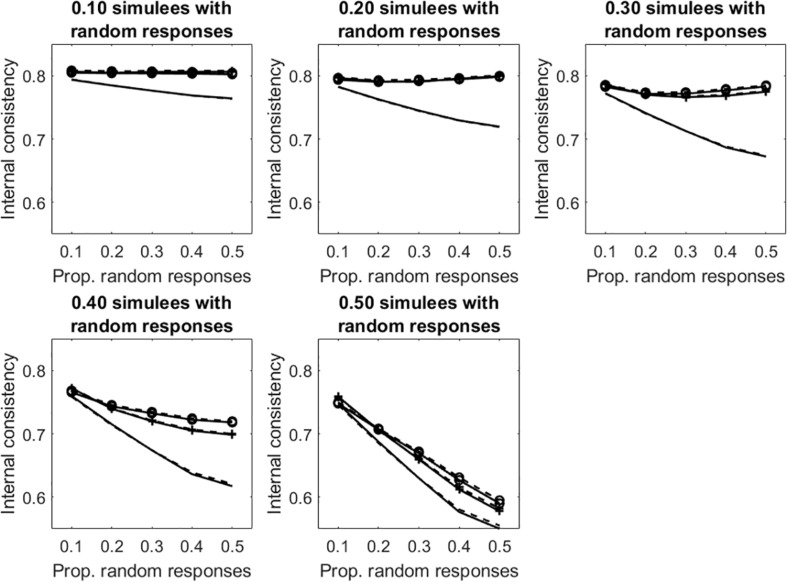
Average internal consistency for the different proportions of simulees with random responses and the different proportions of dichotomous items with random responses. The solid lines represent KR20, the dashed lines represent R. The unmarked lines represented the full, uncleaned data sets. The +-marked lines represent infit-cleaned data sets. The o-marked lines represent the outfit-cleaned data sets.

Figure 6 displays the average internal consistency for the different proportions of simulees with random responses and the different proportions of random responses in the patterns. The solid lines represent KR20, the dashed lines represent R. The unmarked lines represented the uncleaned data sets. The +-marked lines represent the infit-cleaned data sets. The o-marked lines represent the outfit-cleaned data sets. When simulees with random responses represent 10% of the sample, internal consistency obtained on the uncleaned data sets decreases with the increasing of the proportion of random responses in the patterns, whereas that obtained by removing underfitting simulees does not change. Even if the cut-off allows for identifying only a few of the simulees with random responses on 10% of items (sensitivity = 0.30, 0.51 for infit and outfit, respectively; see Table 1), the remaining simulees represent a small part of the sample so that they do not affect internal consistency too much. When the proportion of items with random responses increases to 0.50 (so that the random responses are a substantial threat for internal consistency), almost all of the underfitting simulees are correctly identified and removed (sensitivity = 0.98, 0.99 for infit and outfit, respectively; see Table 1). Similar results are observed when the proportion of simulees with random responses is 0.20 or 0.30. When this proportion is 0.40 or larger, the measures of internal consistency obtained by removing the underfitting simulees decrease with the increase with the proportion of missing data in the patterns. This is due to the fact that, when simulees with random responses become a consistent part of the sample, the cut-off fails in identifying a large part of them (with *p*_sim_ = 0.40, sensitivity ≤ 0.42, 0.55 for infit and outfit, respectively; with *p*_sim_ = 0.50, sensitivity ≤ 0.18, 0.33 for infit and outfit, respectively). Therefore, these simulees remain in the sample and affect internal consistency. Since sensitivity is larger for outfit than for infit, internal consistency obtained by removing simulees on the basis of outfit is never lower than that obtained by removing them on the basis of infit.

**FIGURE 7:**
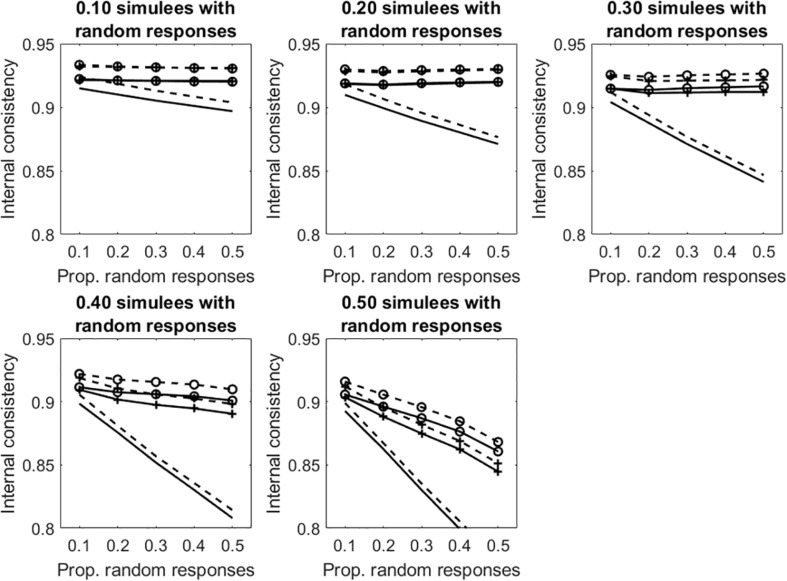
Average internal consistency for the different proportions of simulees with random responses and the different proportions of polytomous items with random responses. The solid lines represent α, the dashed lines represent R. The unmarked lines represented the full, uncleaned data sets. The +-marked lines represent infit-cleaned data sets. The o-marked lines represent the outfit-cleaned data sets.

Similar results are obtained in the tests with polytomous items (see Figure 7 and Table 2).

**TABLE 2:** Sensitivity and specificity of infit and outfit in the tests with polytomous items.

		**Infit**		**Outfit**	
***p*_sim_**	***p*_resp_**	**Sensitivity**	**Specificity**	**Sensitivity**	**Specificity**
0.10	0.10	0.55	0.92	0.69	0.86
0.10	0.20	0.83	0.95	0.90	0.90
0.10	0.30	0.93	0.97	0.97	0.93
0.10	0.40	0.98	0.98	0.99	0.95
0.10	0.50	1.00	0.99	1.00	0.97
0.20	0.10	0.49	0.95	0.64	0.90
0.20	0.20	0.72	0.98	0.84	0.95
0.20	0.30	0.86	0.99	0.93	0.98
0.20	0.40	0.92	0.99	0.97	0.99
0.20	0.50	0.96	1.00	0.98	1.00
0.30	0.10	0.43	0.97	0.58	0.93
0.30	0.20	0.61	0.99	0.75	0.98
0.30	0.30	0.74	1.00	0.85	0.99
0.30	0.40	0.81	1.00	0.90	1.00
0.30	0.50	0.86	1.00	0.93	1.00
0.40	0.10	0.36	0.98	0.53	0.96
0.40	0.20	0.50	0.99	0.67	0.99
0.40	0.30	0.60	1.00	0.75	1.00
0.40	0.40	0.66	1.00	0.79	1.00
0.40	0.50	0.69	1.00	0.80	1.00
0.50	0.10	0.29	0.99	0.48	0.97
0.50	0.20	0.39	1.00	0.59	1.00
0.50	0.30	0.46	1.00	0.63	1.00
0.50	0.40	0.49	1.00	0.63	1.00
0.50	0.50	0.48	1.00	0.61	1.00

*p_*sim*_ = proportion of simulees with random responses; p_*resp*_ = proportion of random item responses. Cut-off for infit and outfit = 1.3.*

### Brief Discussion

Internal consistency decreases with the increasing of random responses in the data set. Two options for dealing with such responses have been presented that are based on infit and outfit statistics. The first option implies using infit and outfit to compute more conservative estimates of internal consistency. In the presented simulations, the measures based on outfit were found to be more conservative than those based on infit.

The second option implies using infit and outfit to detect individuals with random responses. These statistics are a valid tool for this purpose, especially when there are a few individuals with a consistent number of random responses. Under these conditions, infit and outfit allow for correctly detecting almost all of them. When these individuals are removed, the internal consistency computed on the cleaned data sets is similar to the true internal consistency. In the presented simulations, outfit outperformed infit in identifying individuals with random responses. Consequently, the internal consistency obtained on the outfit-cleaned data sets resembled the true internal consistency more than that obtained on the infit-cleaned data sets.

## Overall Discussion

The present work compared classical and modern measures of internal consistency, which were computed on data sets with dichotomous and polytomous items. Classical and modern estimates of internal consistency are largely the same when the score distribution is substantially symmetric, whereas they differ when the score distribution is skewed. R is more conservative than KR20 and α, and prevents test users from believing a test has better measurement characteristics than it actually has. Compared with KR20 and α, R is expected to be a better index of internal consistency as the numerical values are linear rather than non-linear, and the actual average error variance of the sample is used instead of the error variance of an “average” respondent (Wright and Stone, 1999; Smith, 2001).

Internal consistency decreases with the increasing of random responses in the data set. Two options for dealing with such responses have been presented that are based on Rasch-based infit and outfit statistics. The first option implies using infit and outfit to compute a more conservative estimate of internal consistency. The second option implies using infit and outfit to detect individuals with unexpected responses. When there are a few individuals who gave a consistent number of unexpected responses, infit and outfit allow for correctly detecting almost all of them. The response pattern of each of these individuals can be carefully analyzed to try to discover the reason behind the unexpected responses (Has the individual responded randomly? Does he/she belong to a different population?). Once the individuals with random responses are removed, a cleaned data set is obtained that can be used for computing a less biased estimate of internal consistency.

### Limitations and Suggestions for Future Research

In the present study, the data have been simulated under the assumption that the Rasch model was true in the population. Although KR20, α, and R are based on the same measurement model, it is not possible to exclude that the data generating process might have influenced the results. In future studies, the data could be generated using some procedure that puts the different indexes on an equal footing. For instance, the data could be generated from a multivariate normal distribution with the same variance for all items and the same covariance for all pairs of items. Alternatively, they could be generated from a one-factor model with equal factor loadings for all items.

In the present study, Rasch-based R has been shown as an example of modern measure of internal consistency. However, there are other models within modern test theory, which are distinct from Rasch models and pertain to item response theory (IRT). As for the Rasch models, there are several applications of IRT models to the development and validation of measurement scales (see, e.g., Wagner and Harvey, 2006; Thomas, 2011; Zanon et al., 2016; Colledani et al., 2018a, b, 2019a,b). Future studies should investigate the functioning of IRT-based measures of internal consistency, and compare them with classical and Rasch-based measures.

Random responding is only one type of careless responding. Another type of careless responding is identical responding. Individuals with this response behavior may give a certain response (e.g., Strongly agree) to all the items on one page and give the same or another response (e.g., Agree) to all the items on the next page. Future studies should investigate whether infit and outfit statistics allow the identification of individuals with this type of response behavior. Certainly, infit and outfit are unable to detect individuals who choose an *extreme* (minimum or maximum) response option for *all* test items, when there are no reverse-keyed items. Response patterns with extreme scores to all test items always fit the Rasch model perfectly (Linacre, 2019), so infit and outfit are not computed for them. Nevertheless, it is worth noting that these response patterns can be simply identified by looking at the average and standard deviation of the item responses (the former being equal to the minimum or maximum response score; the latter being equal to 0).

## Data Availability Statement

The R scripts used for simulating and analyzing the data will be made available by the authors, without undue reservation, to any qualified researcher.

## Author Contributions

PA, DC, and ER contributed conception and design of the study, manuscript revision, read and approved the submitted version. PA performed the statistical analyses and wrote the first draft of the manuscript.

## Conflict of Interest

The authors declare that the research was conducted in the absence of any commercial or financial relationships that could be construed as a potential conflict of interest.
